# Supplementary material for: Skotomorphogenesis exploits threonine to promote hypocotyl elongation
Source: Quant Plant Biol. 2022 Nov 22;3:e26. doi: 10.1017/qpb.2022.19 (PMC10095960; doi:10.1017/qpb.2022.19)
Supplement: Supplementary file 1 [file S2632882822000194sup001.zip › S2632882822000194sup001.pdf]

Supplementary Material

Skotomorphogenesis exploits threonine to promote hypocotyl elongation

Hiromitsu Tabeta, Higashi Yasuhiro, Okazaki Yozo, Kouji Takano, Kiminori Toyooka, Mayumi Wakazaki, Mayuko Sato, Kazuki Saito, Masami Y Hirai, Ali Ferjani

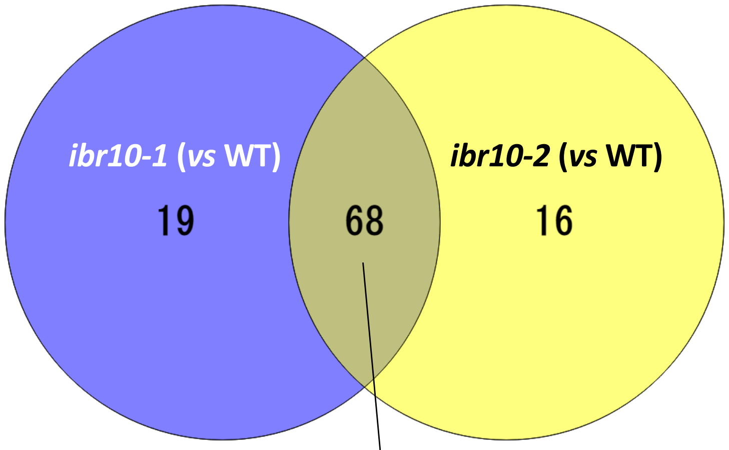

Threonic acid-4TMS	Kynurenine-2TMS
N-Acetylmannosamine-meto-4TMS	5-Oxoproline-2TMS
Isoleucine-2TMS	Shikimic acid-4TMS
Valine-2TMS	Malic acid-3TMS
3-Hydroxybutyric acid-2TMS	Pyruvic acid-meto-TMS
3-Hydroxy-3-methylglutaric acid-3TMS	Glyoxylic acid-meto-TMS
Trehalose-8TMS	Citric acid-4TMS
Adipic acid-2TMS	Docosapentaenoic acid-TMS
Glutaric acid-2TMS	meso-Erythritol-4TMS
Fucose-meto-4TMS	2-Aminopimelic acid-3TMS
Rhamnose-meto-4TMS	Cytidine-4TMS
Sucrose-8TMS	Lactose-meto-8TMS
2-Methyl-3-hydroxybutyric acid-2TMS	Succinic acid-2TMS
Inosine-4TMS	Tartaric acid-4TMS
Glucosamine-5TMS	Galactose-meto-5TMS
Fumaric acid-2TMS	3-Hydroxyisobutyric acid-2TMS
Maltose-meto-8TMS	Arabinose-meto-4TMS
Inositol-6TMS	Hippuric acid-TMS
Xylose-meto-4TMS	Adenine-2TMS
2-Hydroxyglutaric acid-3TMS	Glucono-1,5-lactone-4TMS
5-Aminovaleric acid-2TMS	Glucose-meto-5TMS
Psicose-meto-5TMS	Allose-meto-5TMS
Sorbose-meto-5TMS	Mannose-meto-5TMS
Glyceric acid-3TMS	Ribose-meto-4TMS
Leucine-2TMS	Acetylglycine-2TMS
Methylmalonic acid-2TMS	Threitol-4TMS
Tagatose-meto-5TMS	Mannitol-6TMS
Fructose-meto-5TMS	4-Aminobutyric acid-2TMS
Pyridoxal-meto-2TMS	3-Phenyllactic acid-2TMS
Malonic acid-2TMS	Pyridoxamine-4TMS
Dimethylglycine-TMS	2-Ketoadipic acid-oxime-3TMS
Tyrosine-3TMS	Phosphoric acid-3TMS
N-Acetylneuraminic acid-6TMS	Glycine-3TMS
Lactitol-9TMS	2-Aminoadipic acid-3TMS

Supplementary Figure S1. Metabolic changes in *ibr10* mutants. (In support of Figure 2)

Venn diagram for each mutant line (3 DAI). Numbers represent the metabolites whose contents were significantly changed compared with the WT as determined by Student's *t*-test (n = 6, $P < 0.05$). Factors shown in the black box are significantly different in all mutants compared with the WT. DAI, days after induction of seed germination.

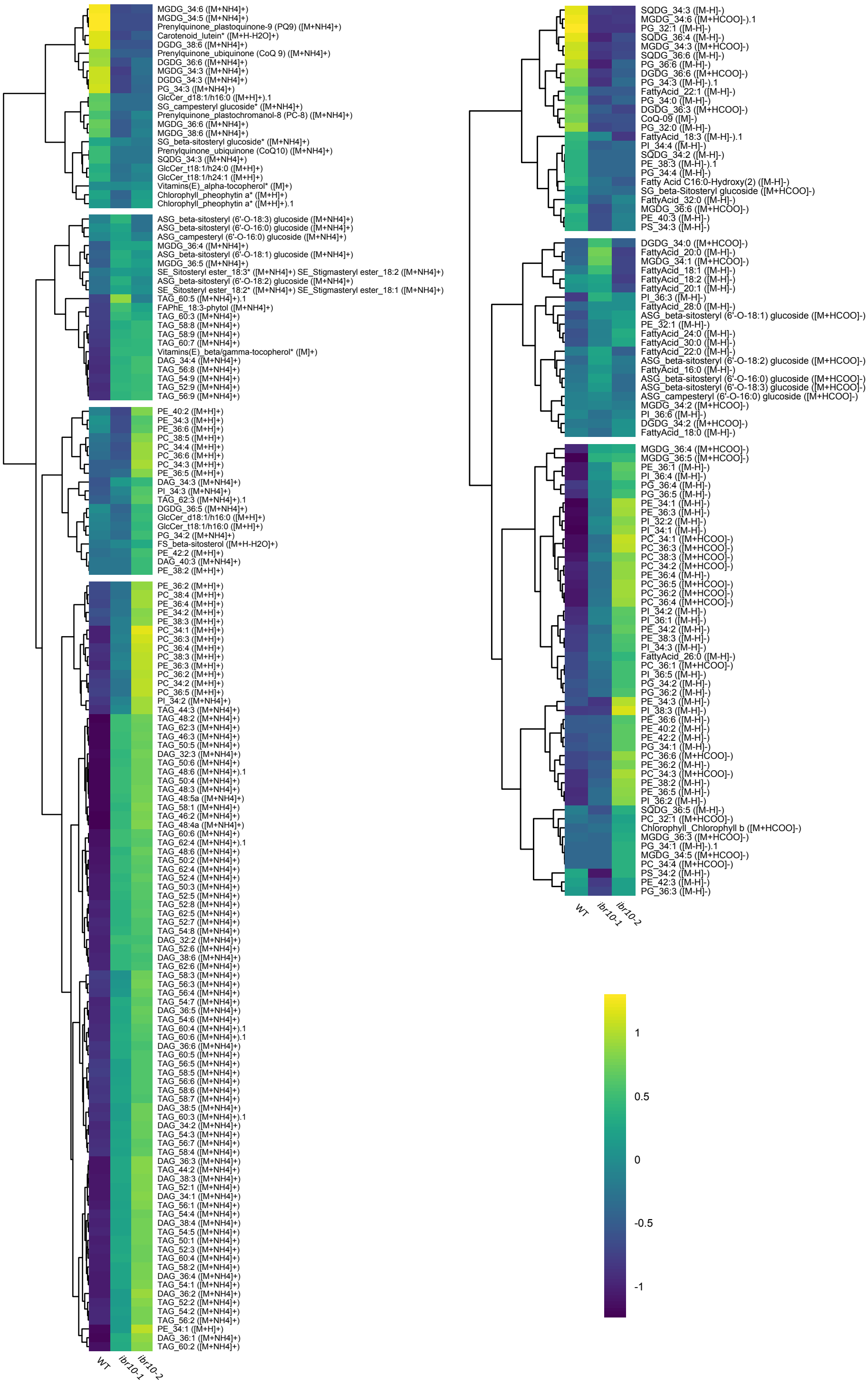

Supplementary Figure S2. LC-MS/MS lipidome analysis of *ibr10* mutants. (In support of Figure 3)

Hierarchical clustering analysis of *ibr10-1* and *ibr10-2* is shown. All 146 metabolites detected by LC-qTOF-MS were categorized into several clusters based on their behavior. The right and left sides show metabolites detected in positive and negative modes, respectively. Data are Z-scores of the average relative contents (n = 6) using 3 DAI etiolated seedlings. DAI, days after induction of seed germination.

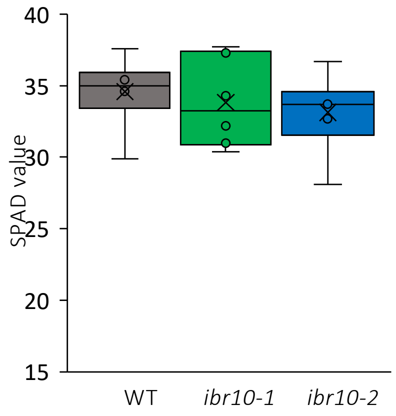

Supplementary Figure S3. SPAD values during the vegetative growth phase. (In support of Figure 4)

The SPAD value of a rosette leaf of *ibr10*. SPAD values were measured at 36 DAS (n = 6, biological replicates). Lower and upper boxes represent the first and third quartiles, respectively, and the line in the boxes represents the median. Each single biological replicate contains 10 technical replicates. DAS, days after seed sowing.

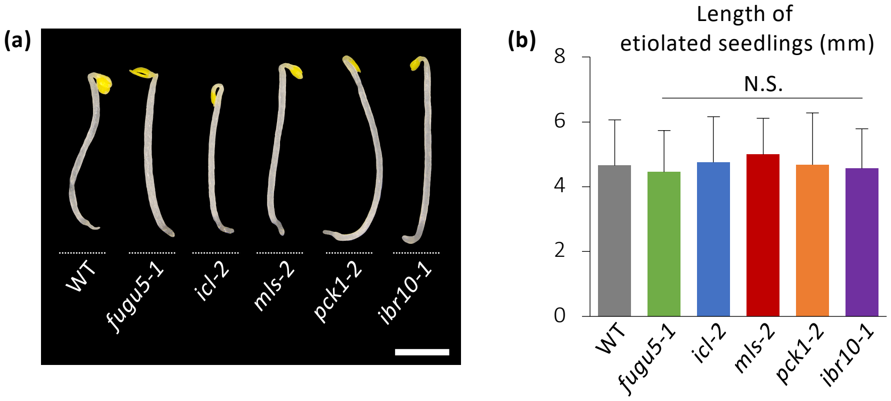

Supplemental Figure S4. Hypocotyl length of etiolated seedlings grown on MS medium with 2% sucrose. (In support of Figure 5A)

Phenotypes of etiolated seedlings grown on MS medium with 2% sucrose. (a) Photographs were taken at 3 DAI. Bar = 2 mm. (b) Data of bar plot are means + SD of 3 DAI etiolated seedlings (*n* = 20). N.S. indicates that mutants have no significant differences compared to the WT (Dunnett's test at $p < 0.05$; R ver. 3.5.1). DAI, day after induction of seed germination.

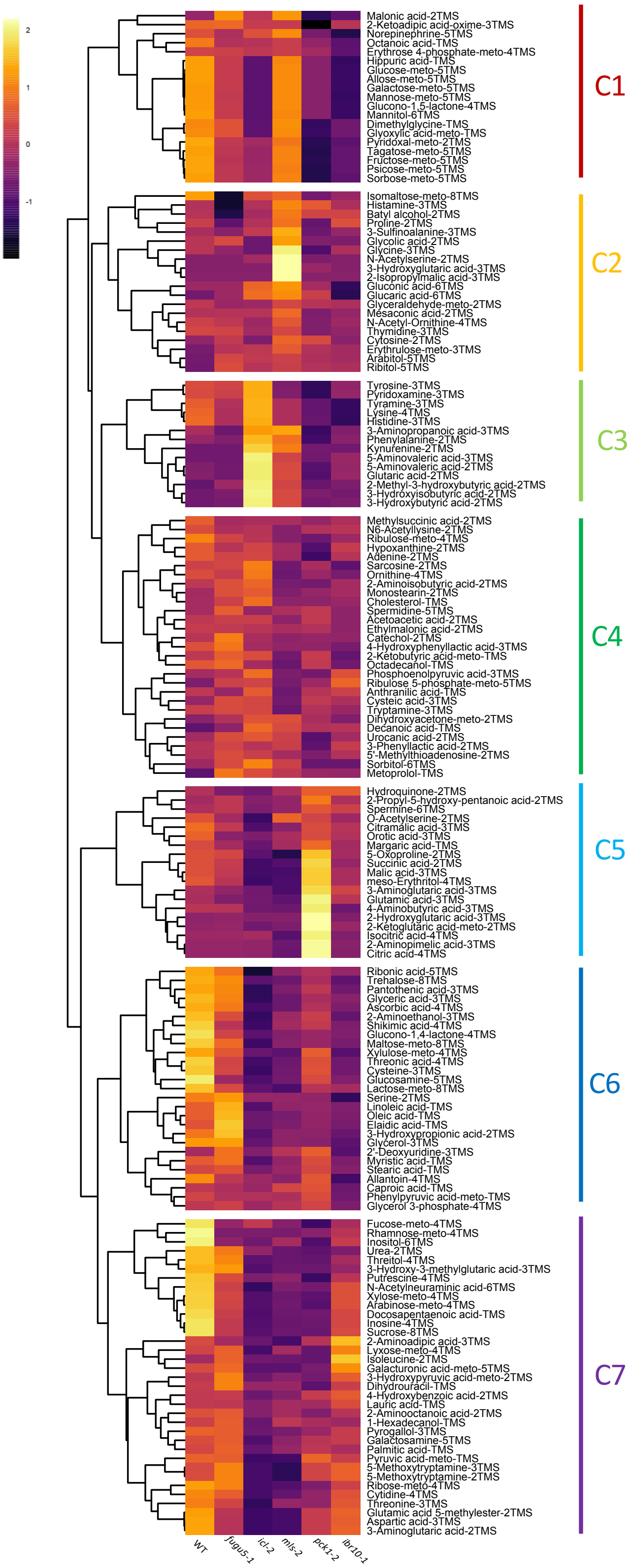

Supplementary Figure S5. Metabolic changes in Class II compensation mutants. (In supports Figure 5)

Hierarchical clustering analysis of Class II compensation-exhibiting mutants is shown. All 163 metabolites detected by GC-QqQ-MS were categorized into seven clusters based on behavior. Data are Z-scores of the average contents relative to that of the quality control sample (*n* = 4). Three DAI etiolated seedlings. DAI, days after induction of seed germination.

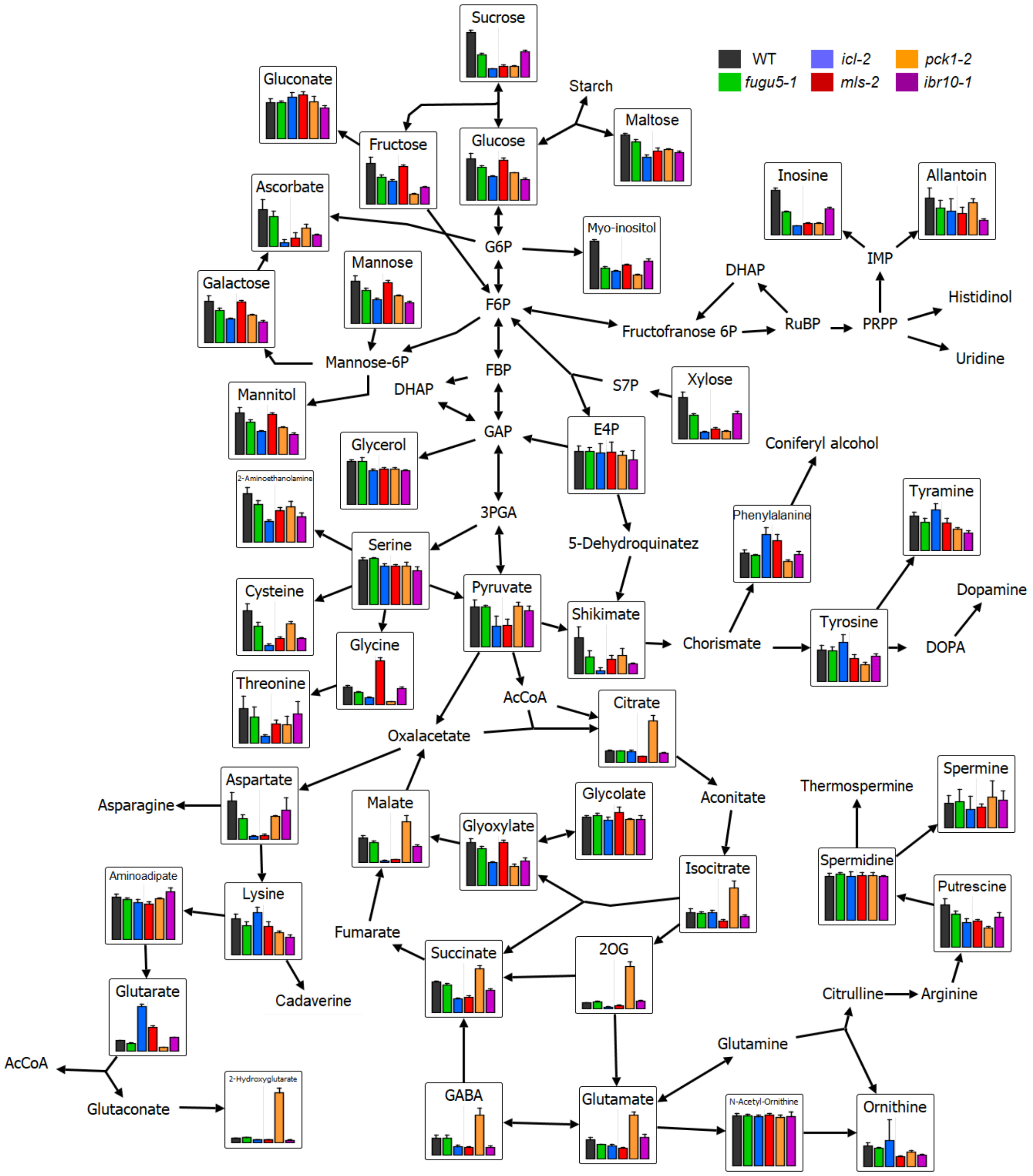

Supplementary Figure S6. Pathway analysis of Class II compensation mutants. (In support of Figure 5)

Pathway analysis of Class II compensation-exhibiting mutants. Primary metabolites related to TAG-to-Suc conversion were plotted on the corresponding metabolic pathway. Data are means + SD (*n* = 4). Three DAI etiolated seedlings. DAI, days after induction of seed germination.

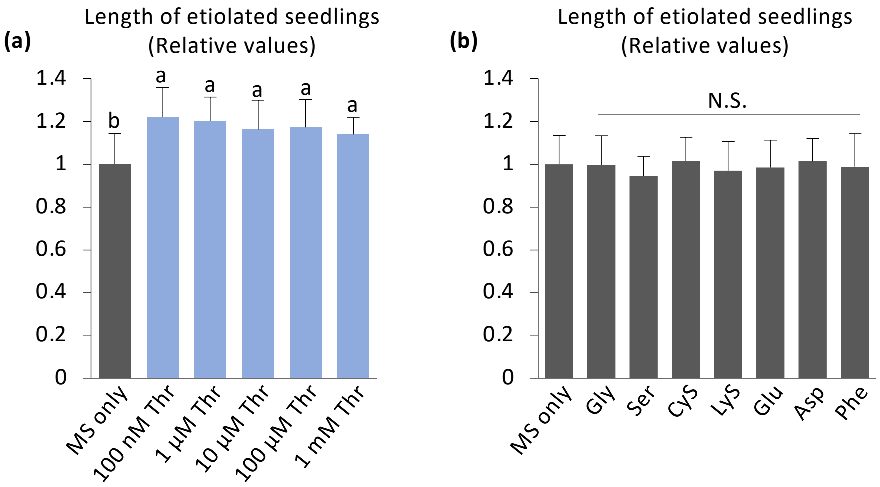

Supplemental Figure S7. Length of etiolated seedlings grown with exogenous amino acids (In support of Figure 5E)

(a) Length of etiolated seedlings grown for 3 DAI on Suc-free MS medium supplied with various concentrations of Thr. Data of bar plot are means + SD (*n* = 20). Letters indicate groups (Tukey's HSD test at $p < 0.01$; R ver. 3.5.1). **(b)** Length of etiolated seedlings grown for 3 DAI on Suc-free MS medium supplied with 100 µM of the indicated amino acids. Data of bar plot are means + SD (*n* = 20). N.S. indicates that mutants did not show statistically significant difference compared to the WT (Dunnett's test at $p < 0.5$; R ver. 3.5.1). DAI, days after induction of seed germination.
